# The Association between Nonalcoholic Fatty Liver Disease and Stroke: Results from the Korean Genome and Epidemiology Study (KoGES)

**DOI:** 10.3390/ijerph17249568

**Published:** 2020-12-21

**Authors:** Yun-Jung Yang, Mi-Hyang Jung, Seok-Hoo Jeong, Yeon-Pyo Hong, Yeong In Kim, Sang Joon An

**Affiliations:** 1 Institute of Biomedical Science, Catholic Kwandong University International St. Mary’s Hospital, Incheon 22711, Korea; yangyj@ish.ac.kr; 2 Cardiovascular Center, Dongtan Sacred Heart Hospital, Hallym University College of Medicine, Hwaseong-Si 18450, Korea; floria0515@gmail.com; 3 Division of Gastroenterology, Department of Internal Medicine, Catholic Kwandong University International St. Mary’s Hospital, Incheon 22711, Korea; ssukoo@naver.com; 4 Department of Preventive Medicine, College of Medicine, Chung-Ang University, Seoul 06974, Korea; hyp026@cau.ac.kr; 5 Department of Neurology, Catholic Kwandong University International St. Mary’s Hospital, Incheon 22711, Korea; nuyikim@ish.ac.kr

**Keywords:** stroke, non-alcoholic fatty liver disease, incidence, risk factors, liver, cohort studies

## Abstract

(1) Background: Non-alcoholic fatty liver disease (NAFLD) is associated with various cardiometabolic diseases. However, the association between NAFLD and stroke is not well known. The purpose of our study is to reveal the relationship between NAFLD and Stroke incidence. (2) Methods: Using data from a Korean prospective cohort study, we excluded participants with heavy alcohol consumption and a history of stroke; hence, 7964 adults aged 40–69 years were included in this study. According to their fatty liver index (FLI), participants were divided into three groups: <30 (*n* = 4550, non-NAFLD), 30–59.9 (*n* = 2229, intermediate), and ≥60 (*n* = 1185, NAFLD). The incidence of stroke according to the degree of FLI was evaluated using the Cox proportional hazard model. (3) Results: During the 12-year follow-up period, 168 strokes occurred. A graded association between NAFLD and stroke incidence was observed, i.e., 1.7% (*n* = 76), 2.5% (*n* = 56), and 3.0% (*n* = 36) for non-NAFLD, intermediate, and NAFLD FLI groups, respectively. After adjusting for confounding variables and compared to the risk of stroke in the non-NAFLD group, the risk of stroke in the NAFLD group was the highest (hazard ratio [HR]: 1.98, 95% confidence interval [CI]: 1.17–3.34), followed by the risk of stroke in the intermediate group (HR: 1.41, 95% CI: 0.94–2.21) (*p* for trend < 0.001). However, the level of aspartate aminotransferase, alanine aminotransferase, or gamma-glutamyltransferase alone did not show any significant association with stroke. (4) Conclusions: This study demonstrated that the risk of stroke incidence gradually increased with the degree of FLI. Individuals with NAFLD should be properly counseled and monitored for risk for stroke.

## 1. Introduction

Stroke is a significant health burden worldwide [1,2,3]. The prevalence of stroke, the third-largest cause of death in Korea, is increasing rapidly [4]. Every year, there are 105,000 new or recurrent stroke cases in Korea. An average of one new stroke occurs every 5 min [5]. Stroke is a heterogeneous disease arising from several distinct underlying pathologies [6]. Therefore, the mechanisms and risk factors of stroke vary widely. Classical risk factors include nonmodifiable characteristics, such as age and ethnicity, and modifiable risk factors, such as coronary heart disease, atrial fibrillation, diabetes mellitus, hypertension, smoking, dyslipidemia, and obesity [7,8].

Non-alcoholic fatty liver disease (NAFLD) is characterized by the presence of significant lipid accumulation in the liver parenchyma without excessive alcohol ingestion or any other liver disease [9]. NAFLD is the most common chronic liver disease and affects approximately 34–46% of the population [10]. NAFLD has been known to be associated with various cardiovascular diseases, including type 2 diabetes mellitus and obesity, and emerging risk factors related to subclinical atherosclerosis and reduced adiponectin levels [11]. However, the role of NAFLD in stroke is not conclusive and results have varied across studies [12,13,14,15]. Currently, limited data are available for many Asians including Koreans [16]. Moreover, Asians have different stroke characteristics from Western populations with a higher prevalence of hemorrhagic stroke than ischemic stroke [7] and different metabolic profiles with worse insulin resistance and more body fat with the same body mass index [17]. Previous data estimating the role of NAFLD in stroke risk are inconsistent and inconclusive, probably because of the diverse study populations [18]. Therefore, this study aimed to characterize the role of NAFLD in the risk of stroke in middle-aged adults based on a prospective cohort study in Korea.

## 2. Materials and Methods

### 2.1. Study Subjects

This study used data from the Ansung–Ansan cohort, which is part of the Korean Genome and Epidemiology Study (KoGES). The design and survey methods of the KoGES have been previously described [19]. Briefly, the Ansung–Ansan cohort has existed since 2001 including 10,030 adults aged 40 to 69 living in the area. They have undergone face-to-face surveys and physical examinations. The details of this cohort have been described in a previous study [19].

**Figure 1:**
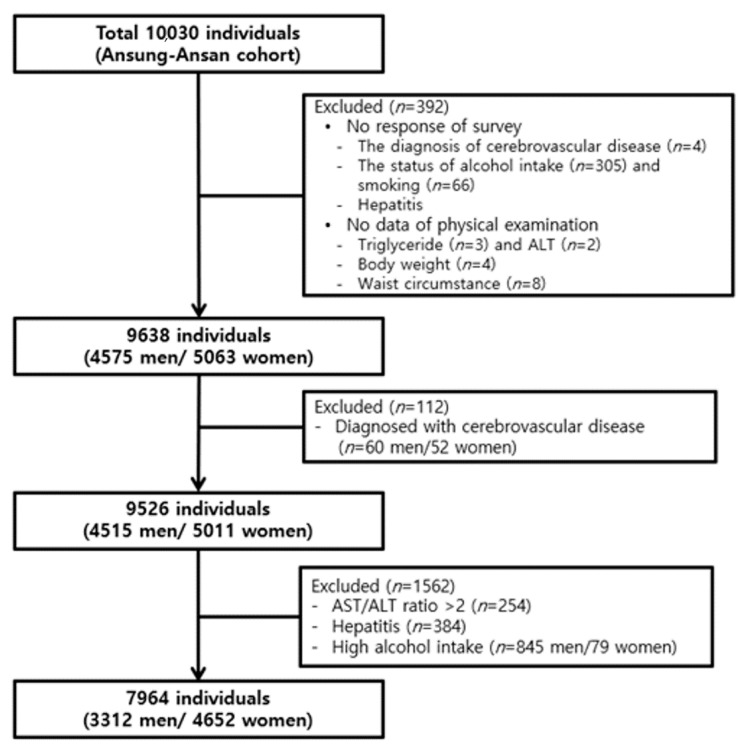
The study population in the present study was obtained from the Ansung–Ansan cohort study.

Participants with missing data regarding the diagnosis of cerebrovascular disease (*n* = 4), alcohol consumption (*n* = 305), smoking status (*n* = 66), triglyceride, aspartate aminotransferase (AST), alanine aminotransferase (ALT), body weight and waist circumstance (*n* = 17) were excluded. Subjects with pre-existing cerebrovascular disease (*n* = 112; 60 men, 52 women) were further excluded. Among the remaining subjects, 1562 were excluded because of AST/ALT ratio > 2, as this can be an indicator of alcoholic liver disease (*n* = 254), self-reported history of hepatitis (*n* = 384), or alcohol intake amount >30 g/day in men and >20 g/day in women (*n* = 845 men and *n* = 79 women). Finally, 7964 participants (3312 men and 4652 women) were included in this analysis (Figure 1). The study protocol was approved by the Korea Centers for Disease Control and Prevention Institutional Review Board and by the Institute Review Board at the Catholic Kwandong University International St. Mary’s Hospital (IS18EISI0071).

### 2.2. Data Collection

The demographic and behavioral information including age, sex, physical activity, smoking and drinking status, medical history, and the use of medication was obtained from a face-to-face survey performed by a trained interviewer. Age was divided into two groups based on the population median. Smoking and alcohol status was classified as never, former, and current. The intensity of physical activity was categorized according to the quartile of the metabolic equivalent of task (MET) values. Cancer was defined as a self-reported history of lung cancer, stomach cancer, liver cancer, colon cancer, pancreatic cancer, uterine cancer, breast cancer, thyroid cancer, prostate cancer, or gallbladder cancer.

Bodyweight, height, and waist circumstance were measured following the standard methods. Body mass index (BMI, kg/m^2^) was calculated as body weight divided by height squared. BMI was classified as normal and obese according to the World Health Organization cut-off values for Asian adults: normal weight as BMI 18.5–24.9 kg/m^2^, underweight as BMI < 18.5 kg/m^2^, moderate obesity as BMI 25.0–29.9 kg/m^2^, and severe obesity as BMI ≥ 30 kg/m^2^. Hypertension was defined as self-reported history of hypertension, use of antihypertensive drugs, or systolic blood pressure ≥ 140 mmHg or diastolic blood pressure (DBP) ≥ 90 mmHg. Diabetes mellitus was defined as a self-reported history of diabetes mellitus, use of the antidiabetic drug, or fasting glucose level ≥ 126 mg/dL. Hyperlipidemia was defined as a self-reported history of hyperlipidemia, use of anti-hyperlipidemia drug, triglyceride ≥ 240 mg/dL, high-density lipoprotein cholesterol ≤ 40 mg/dL, or low-density lipoprotein cholesterol ≥ 160 mg/dL. Cardiovascular disease was defined as a self-reported history of cardiovascular disease. The use of anti-hyperlipidemia drug was defined as past or current use of an anti-hyperlipidemia drug in self-reported history. The occurrence of stroke was determined using a questionnaire that asked participants whether they had ever been diagnosed with a stroke by a doctor. Stroke subtype (e.g., ischemic, hemorrhagic) was not specified in the questionnaire. Data of the following biochemical parameters were collected: serum triglyceride (TG), gamma-glutamyltransferase (GGT), ALT, and AST.

### 2.3. Measurement of Fatty Liver Index (FLI) and Definition of NAFLD

The FLI was calculated to determine the NAFLD status. BMI, GGT, waist circumstance, and triglycerides were used to calculate FLI according to the following formula:

$$FLI=\frac{e^{0.953\times \log(TG)+0.139\times BMI+0.718\times \log(GGT)+0.053\times waistcircumference-15.745}}{1-e^{0.953\times \log(TG)+0.139\times BMI+0.718\times \log(GGT)+0.053\times waistcircumference-15.745}}\times 100$$

with TG measured in mmol/L, GGT in U/L, and waist circumference in ftcm. According to a previous study [20], FLI ≥ 60 was considered as representative of liver steatosis.

Subjects were classified into three groups based on the FLI score; FLI < 30 was defined as non-NAFLD, FLI 30–59.9 was defined as intermediate, and FLI ≥ 60 was defined as NAFLD.

### 2.4. Statistical Analysis

Data are expressed as the mean and standard error (continuous) or as frequencies and percentages (categorical). Comparisons between groups were performed using the *t*-test (continuous variable) or χ^2^ test (categorical). Survival time was defined as the time between the baseline and onset of the outcome or final follow-up. Patients who were lost to follow-up or had died were censored. The Cox proportional hazard model was used to estimate the association between FLI and incidence of stroke after adjustment for confounding variables. The incidence time was calculated by subtracting the date of initial participation from the final participation date. Multivariate analysis adjusted for FLI, age, sex, and comorbidities with robust sandwich estimators. Model 1 either adjusted for or was stratified by non-modifiable risk factors such as age and sex. Model 2 was additionally adjusted for well-known and proven modifiable risk factors such as hypertension, diabetes mellitus, hyperlipidemia, cardiovascular disease, smoking, alcohol status, and body mass index. Model 3 was additionally adjusted for other factors such as the metabolic equivalent of task, cancer, hyperlipidemia drug, and antihypertension drug. Analyzed results are expressed as hazard ratio (HR) and 95% confidence interval (CI). Data analysis was performed using STATA (version 15.0 StataCorp LP, College Station, TX, USA). *p*-values less than 0.05 were considered significant.

## 3. Results

### 3.1. Baseline Characteristics

The baseline characteristics of participants grouped according to their FLI scores are shown in Table 1. This study included 4550, 2229, and 1185 participants in the non-NAFLD, intermediate, and NAFLD groups, respectively, and the mean ± standard error age was 51.67 ± 0.13, 53.86 ± 0.18, and 53.06 ± 0.25 years, respectively. Men were more predominant in the NAFLD group (*n* = 708, 59.75%) than in the intermediate (*n* = 1043, 46.79%) and non-NAFLD (*n* = 1056, 34.3%) groups. Moreover, compared with participants in the non-NAFLD and intermediate groups, participants in the NAFLD group had higher alcohol consumption (*n* = 614, 51.8%) and smoking (*n* = 378, 31.9%) rates and incidences of hypertension (*n* = 716, 60.4%), diabetes mellitus (*n* = 200, 16.9%), hyperlipidemia (*n* = 859, 72.5%) as well as a higher BMI (28.05 ± 0.09) (all *p* < 0.001).

### 3.2. Incidence and Hazard Ratios of Stroke According to the FLI Group

Table 2 shows the risk of stroke incidence according to the baseline FLI group. Around 1.67% (76/4550) of the non-NAFLD, 2.51% (56/2229) of the intermediate, and 3.03% (36/1185) of the NAFLD group developed stroke. The risk of developing stroke was higher in the NAFLD group (HR = 1.92, 95% CI = 1.29–2.85) than in the intermediate (HR = 1.53, 95% CI = 1.08–2.16) and non-NAFLD group (HR = 1) in the non-adjusted model. With the FLI < 30 group as the reference, the hazard ratios for stroke incidence increased in both groups after adjusting for confounding variables. After adjustment for age and sex (Model 1), NAFLD was associated with an increased risk of developing stroke (HR = 1.64, 95% CI = 1.10–2.46). In Model 2 (adjustment for hypertension, diabetes mellitus, hyperlipidemia, cardiovascular disease, smoking, alcohol status, and BMI) and Model 3 (Model 2 with additional adjustment for MET, cancer, anti-hyperlipidemic drug use, and antihypertensive drug use), the HRs (95% CI) for stroke incidence in the NAFLD group was 1.97 (1.17–3.32) and 1.98 (1.17–3.34), respectively, compared to that in the non-NAFLD group (*p* for trend < 0.001).

**Figure 2:**
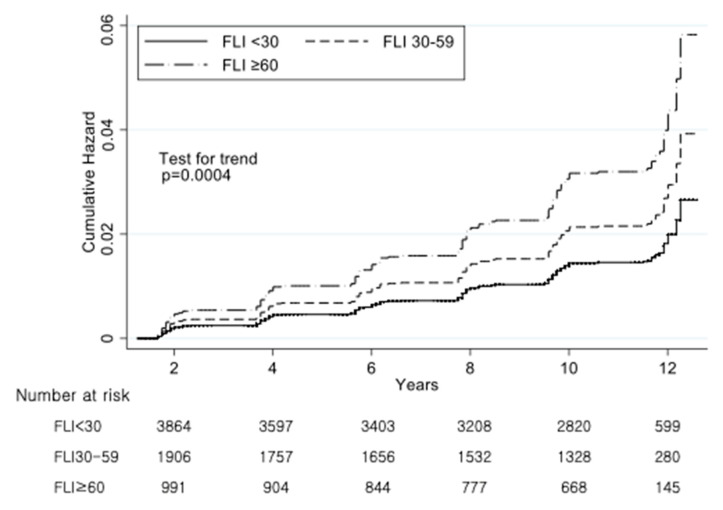
Adjusted cumulative hazard curves for the incidence of cerebrovascular diseases according to the FLI group. Adjusted for age, sex, hypertension, diabetes mellitus, hyperlipidemia, cardiovascular disease, smoking and alcohol status, body mass index (BMI), metabolic equivalent of task (MET), cancer, hyperlipidemia drug, antihypertension drug, aspartate aminotransferase (AST), alanine aminotransferase (ALT), γ-glutamyl transpeptidase (GTP). Estimated by Cox’s proportional hazard regression.

Figure 2 showed the cumulative incidence of stroke in each FLI group. The incidence rate and HRs of stroke were adjusted for age, sex, hypertension, diabetes mellitus, hyperlipidemia, cardiovascular disease, smoking and alcohol status, BMI, MET, cancer, anti-hyperlipidemic drug use, antihypertensive drug use, AST, ALT, and GGT.

### 3.3. Incidence and Hazard Ratios of Stroke According to AST, ALT, and GGT

**Figure 3:**
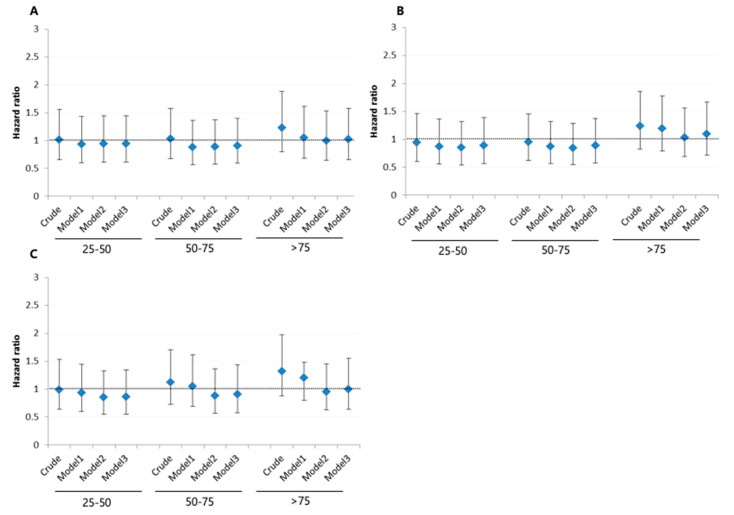
Hazard ratio (95% confidence interval) for the incident of cerebrovascular diseases according to the (**A**) AST, (**B**) ALT, and (**C**) gamma-glutamyltransferase (GGT). Model 1: sex, age. Model 2: model 1 + hypertension, diabetes mellitus, hyperlipidemia, cardiovascular disease, smoking and alcohol status, and body mass index. Model 3: model 2 + metabolic equivalent of task, cancer, hyperlipidemia drug, antihypertension drug, and the metabolic equivalent of task. Estimated from Cox’s proportional hazard regression model.

Figure 3 shows the HR for stroke by quartiles of AST, ALT, and GGT levels. In the analysis of the crude to fully adjusted model (Model 3), the highest quartile was not associated with an increased risk of developing stroke for AST (HR = 1.02, 95% CI = 0.66–1.58), ALT (HR = 1.09, 95% CI = 0.72–1.67), or GGT (HR = 1.00, 95% CI = 0.64–1.55) (Supplementary Table S1: Hazard ratio (95% confidence interval) for the incident of stroke according to the AST, ALT, and GGT groups.).

## 4. Discussions

This study showed the association between NAFLD, ALT, AST, GGT, and stroke incidence. NAFLD as defined by FLI increased the risk of developing future stroke. Stroke developed in 1.67%, 2.51%, and 3.03% in the non-NAFLD, intermediate, and NAFLD groups, respectively. Those with the highest FLI scores (>60) had an approximately two times higher risk of developing stroke (adjusted HR 1.98, 95% CI = 1.17–3.34) relative to the group with the lowest FLI (<30). In addition, AST, ALT, and GGT levels showed no meaningful statistical association.

A previous Korean study [21] revealed that NAFLD defined based on ultrasonography was associated with developing lacunar infarctions in non-obese patients. Furthermore, Wannamethee [22], in a larger prospective study in London, found that NAFLD with lower or moderate cardiovascular risk was independently correlated with a higher risk of ischemic stroke. In another meta-analysis of nine case-control and cohort studies, NAFLD was independently associated with 2.3 times higher risk of ischemic stroke [13].

Although many studies have reported positive associations between NAFLD and stroke, they differed according to age and sex. Kunutsor et al. suggested an inverse association between NAFLD and FLI in older participants (>50 years) and a positive association between NAFLD and FLI in younger participants [23]. In a case-cohort study, Alexander et al. showed that FLI > 60 was associated with a lower risk of stroke in men than in women [18].

Many studies regarding the association between NAFLD and severity or outcome of stroke, rather than the relationship between NAFLD and stroke incidence or risk have been conducted. Patients with NAFLD had more severe strokes, as assessed using the National Institutes of Health Stroke Scale (NIHSS) at admission, and worse functional outcomes, as assessed using the modified Rankin Scale at discharge [12]. However, Baik et al. showed the paradoxical protective effect of NAFLD on functional outcomes and severity after ischemic strokes [16]. Patients with NAFLD had lower NIHSS scores and more favorable functional outcomes [16]. A recent study by Sheng et al. found no relationship between NAFLD and unfavorable outcomes after intracranial hemorrhage [15]. Another study by Li et al. demonstrated a positive association between NAFLD and severity and progression of brain stem infarction [24].

In our study, although NAFLD was associated with stroke development, liver biomarkers (AST, ALT, and GGT) were not associated with stroke development. In a case-cohort study, Alexander et al. showed the hazard ratio of stroke by quintiles of each biomarker, compared with the 1st quintile, and the hazard ratio per SD of each log-transformed marker. There was a strong inverse association of ALT with ischemic stroke in men, but not in women. Higher GGT was associated with reduced stroke risk in men, but no association in women [18]. In a small case-control study by Ying et al. [25], ALT and AST levels were independently associated with three times increased odds ratio for ischemic stroke. In the case-control EUROSTROKE study [26], performed in Finland, the Netherlands, and the United Kingdom, GGT levels were strongly associated with ischemic stroke risk in patients without type 2 diabetes mellitus. In a Korean prospective study, ALT was a predictor of intracerebral hemorrhage but not cerebral infarction [27]. A German case-cohort study found no association between overall stroke and ALT in a middle-aged population but found an association between ischemic stroke and GGT [28]. The British Women’s Heart and Health Study revealed that ALT levels were not associated with stroke in women [29].

The reason why there is a difference in the association with stroke between NAFLD defined by FLI and liver biomarkers is that NAFLD and liver biomarkers have different significances. NAFLD comprises a wide histological spectrum ranging from fat accumulation in hepatocytes without inflammation or fibrosis (simple hepatic steatosis) to hepatic steatosis with necroinflammatory elements (steatohepatitis) [30]. The presence of fibrosis and inflammation is termed non-alcoholic steatohepatitis, which may eventually progress to cirrhosis in up to 20% of patients [31]. Liver dysfunction caused by NAFLD can contribute to thrombotic vascular disease by affecting the synthesis of lipoproteins [32], coagulation proteins [33], and inflammation-related factors [34]. However, elevated liver enzymes do not suggest a correlation between the level of liver injury, fibrosis, or inflammation; therefore, elevated liver enzymes may not be related to the abnormal mechanisms of vasculopathy [35]. ALT is found in the cytosol of hepatocytes where it transfers amino groups, although the major site where AST is found in the mitochondria [36]. Amino group transfer from alanine to ketoglutarate is catalyzed by ALT. Even though ALT is mostly specific to the liver, it can be found in the blood during muscle injury or inflammation [37]. In addition, GGT is less specific to the liver and has been used as a marker of biliary disease and alcoholic intake [38]. Because of these differences, NAFLD as defined by FLI was identified as a potential risk factor for stroke in this study, but the liver index was inappropriate. FLI, which is obtained through TG and BMI, suggests that NAFLD is associated with whole-body obesity and aberration of lipid levels rather than liver disease only and is therefore also associated with cardio and cerebrovascular abnormalities [39,40].

The following are the key strengths of the present study. This is a well-characterized prospective study with a large cohort of participants; it is geographically dispersed in rural and urban areas, and a 12-year follow-up was conducted. Furthermore, it is the first population-based prospective study to explore the potential role of NAFLD in developing stroke in a healthy middle-aged population of Korea.

This study had some limitations. First, our study used a surrogate marker for NAFLD. The gold standard for the clinical diagnosis of NAFLD is liver biopsy, which is not appropriate for epidemiological studies. Although FLI compares well with ultrasound-based determination of fatty liver, it is less accurate than liver biopsy or magnetic resonance imaging for identifying and grading liver steatosis [41]. In support of its use, we saw strong correlations between FLI and components of metabolic syndrome and a higher prevalence of NAFLD in men than in women, which were similar to findings that used an imaging-based NAFLD assessment [42,43]. Second, stroke was not divided into ischemic and hemorrhagic strokes because the occurrence of a stroke was investigated using a questionnaire. Because hemorrhagic strokes leave severe dysfunction, the participants who remained in the cohort and were able to be monitored would have suffered an ischemic stroke. Third, recall bias may have occurred because the KoGES data were based on questionnaires.

## 5. Conclusions

This study demonstrated that the risk of stroke incidence gradually increased with an increase in FLI. Individuals with NAFLD should be properly counseled and monitored for risk for stroke.

## Figures and Tables

**Table 1:** Baseline characteristics of the study participants according to fatty liver index (FLI) score.

Variables	FLI Score			*p*-Value
	<30	30–59	≥60	
Participants, *n*	4550	2229	1185	
Gender, *n* (%)				<0.001
Men	1561 (34.3)	1043 (46.79)	708 (59.75)	
Women	2989 (65.7)	1186 (53.21)	477 (40.25)	
Age, y	51.67 ± 0.13	53.86 ± 0.18	53.06 ± 0.25	<0.001
BMI, kg/m^2^	23.04 ± 0.04	26.02 ± 0.04	28.05 ± 0.09	<0.001
Drinking Status, *n*(%)				<0.001
Never	2615 (57.5)	1131 (50.7)	469 (39.6)	
Former	287 (6.3)	189 (8.5)	102 (8.6)	
Current	1648 (36.2)	909 (40.8)	614 (51.8)	
Smoking Status, *n*(%)				<0.001
Never	3231 (71.0)	1346 (60.4)	563 (47.5)	
Former	516 (11.3)	353 (15.8)	244 (20.6)	
Current	803 (17.7)	530 (23.8)	378 (31.9)	
Physical activity, MET-h/week	23.25 ± 0.22	22.85 ± 0.32	22.52 ± 0.44	0.267
AST	25.91 ± 0.12	29.31 ± 0.38	35.99 ± 0.78	<0.001
ALT	21.44 ± 0.15	30.26 ± 0.55	43.20 ± 1.45	<0.001
γ-GT	16.38 ± 0.16	33.31 ± 0.73	74.41 ± 3.60	<0.001
Comorbidity, *n*(%)				
Hypertension	1310 (28.9)	1107 (49.7)	716 (60.4)	<0.001
Diabetes mellitus	228 (5.0)	255 (11.4)	200 (16.9)	<0.001
Hyperlipidemia	1668 (36.7)	1367 (61.3)	859 (72.5)	<0.001
Coronary artery disease	23 (0.51)	29 (1.3)	13 (1.1)	0.001
Cancer	59 (1.3)	15 (0.7)	12 (1.0)	0.064

FLI: fatty liver index. Values are presented as mean ± standard error.

**Table 2:** Hazard ratio (95% confidence interval) for the incident of stroke according to the FLI groups.

	FLI			*p*-Value
	<30 (*n* = 4550)	30–59.9 (*n* = 2229)	≥60 (*n* = 1185)	
**Incident stroke case (** ***n*** **, %)**	76 (1.67)	56 (2.51)	36 (3.03)	0.0004
**Crude Hazard ratio**	1.00	1.53 (1.08–2.16)	1.92 (1.29–2.85)	0.0025
**Model 1**	1.00	1.31 (0.92–1.86)	1.64 (1.10–2.46)	<0.001
**Model 2**	1.00	1.42 (0.95–2.14)	1.97 (1.17–3.32)	<0.001
**Model 3**	1.00	1.41 (0.94–2.21)	1.98 (1.17–3.34)	<0.001

Model 1: sex, age. Model 2: model 1 + hypertension, diabetes mellitus, hyperlipidemia, cardiovascular disease, smoking and alcohol status, and body mass index. Model 3: model 2 + metabolic equivalent of task, cancer, hyperlipidemia drug, and antihypertension drug. Estimated from Cox’s proportional hazard regression model. FLI: fatty liver index.

## Supplementary Materials

The following are available online at https://www.mdpi.com/1660-4601/17/24/9568/s1, Table S1: Hazard ratio (95% confidence interval) for the incident of stroke according to the AST, ALT, and GGT groups.
