# Supplementary material for: The Association between Nonalcoholic Fatty Liver Disease and Stroke: Results from the Korean Genome and Epidemiology Study (KoGES)
Source: Int J Environ Res Public Health. 2020 Dec 21;17(24):9568. doi: 10.3390/ijerph17249568 (PMC7765788; doi:10.3390/ijerph17249568)
Supplement: Supplementary file 1 [file ijerph-17-09568-s001.pdf]

Supplementary Table1: Hazard ratio (95% confidence interval) for the incident of stroke according to the AST, ALT, and GGT groups.

	AST (%)			
	**<25**	**25-50**	**50-75**	**>75**
**Incident stroke case (n, %)**				
**Crude Hazard ratio**	1.00	1.01 (0.66-1.56)	1.03 (0.67-1.58)	1.23 (0.80-1.88)
**Model 1**	1.00	0.93 (0.60-1.43)	0.88 (0.57-1.36)	1.05 (0.68-1.61)
**Model 2**	1.00	0.94 (0.61-1.44)	0.89 (0.58-1.37)	1.00 (0.65-1.53)
**Model 3**	1.00	0.94 (0.61-1.44)	0.91 (0.59-1.40)	1.02 (0.66-1.58)

	ALT (%)			
	**<25**	**25-50**	**50-75**	**>75**
**Incident stroke case (n, %)**				
**Crude Hazard ratio**	1.00	0.94 (0.60-1.46)	0.95 (0.62-1.45)	1.24 (0.83-1.85)
**Model 1**	1.00	0.87 (0.56-1.36)	0.87 (0.57-1.32)	1.19 (0.79-1.77)
**Model 2**	1.00	0.85 (0.54-1.32)	0.84 (0.55-1.28)	1.03 (0.69-1.56)
**Model 3**	1.00	0.89 (0.57-1.39)	0.89 (0.58-1.37)	1.09 (0.72-1.67)

	GGT (%)			
	**<25**	**25-50**	**50-75**	**>75**
**Incident stroke case (n, %)**				
**Crude Hazard ratio**	1.00	0.99 (0.64-1.53)	1.12 (0.73-1.70)	1.32 (0.88-1.97)
**Model 1**	1.00	0.93 (0.60-1.44)	1.05 (0.69-1.61)	1.20 (0.80-1.48)
**Model 2**	1.00	0.85 (0.55-1.33)	0.88 (0.57-1.36)	0.95 (0.63-1.45)
**Model 3**	1.00	0.86 (0.55-1.34)	0.91 (0.58-1.43)	1.00 (0.64-1.55)

Alanine aminotransferase (AST), Alanine aminotransferase (ALT), γ-glutamyl transpeptidase (GGT)

Model 1: sex, age.

Model 2: model 1 + hypertension, diabetes mellitus, hyperlipidemia, cardiovascular disease, smoking and alcohol status, and body mass index.

Model 3: model 2 + metabolic equivalent of task, cancer, hyperlipidemia drug, and antihypertension drug.

Estimated from Cox's proportional hazard regression model.

AST: Alanine aminotransferase, ALT: Alanine aminotransferase, GGT: γ-glutamyl transpeptidase
